# Prosthetic heart valve selection in women of childbearing age with acquired heart disease: a case report

**DOI:** 10.1186/s13256-016-0821-y

**Published:** 2016-03-08

**Authors:** Leonid Barbarash, Natalya Rutkovskaya, Olga Barbarash, Yuri Odarenko, Alexander Stasev, Evgenya Uchasova

**Affiliations:** Federal State Budgetary Institution Research Institute for Complex Issues of Cardiovascular Diseases, Sosnovy Blvd., 6, Kemerovo, 650002 Russian Federation

**Keywords:** Bioprosthesis, Childbirth, Fertile women, Pregnancy

## Abstract

**Background:**

The problem of prosthetic heart valve selection in fertile women with acquired heart defects remains crucial in modern cardiology. Mechanical heart valves require lifelong indirect anticoagulant therapy, which has significant fetal toxicity and is unacceptable for women planning pregnancy. Bioprosthetic heart valves are the best choice for fertile women; however, their durability is limited, and reoperations are required.

**Case presentation:**

We describe the clinical case of a 21-year-old Russian woman with infectious endocarditis who underwent heart valve replacement with an epoxy-treated mitral valve prosthesis.

**Conclusions:**

Epoxy-treated bioprosthetic heart valves can be used without long-term anticoagulant therapy because of their optimal hemodynamic functional parameters. Moreover, their high thromboresistance and resistance to infection improve patients’ quality of life in their late postoperative period. We recommend these valves both in older persons and in young patients including women who are planning pregnancy.

## Background

Acquired heart disease (AHD) is currently the most common cardiac disease among young adults in Russia, with infectious and rheumatic valvular lesions predominating [1]. In contrast, European hospitals report a higher prevalence of degenerative valve disease, and thus their heart valve replacement patients tend to be older persons [2].

Heart valve replacement surgery significantly prolongs life expectancy and improves quality of life in patients with AHD. There are two basic types of prosthetic heart valves used in current clinical practice: mechanical and bioprosthetic. Deciding which valve to use requires careful consideration of the specific advantages and disadvantages of the valve types and integration of this knowledge into the clinical characteristics and personal preferences of the individual patient [3].

European clinics have solved the problem of selecting prosthetic heart valves for patients with AHD by implementing the American College of Cardiology/American Heart Association (AHA) 2006 guidelines and the European Society of Cardiology (ESC) 2007 guidelines [3]. Unfortunately, European and American guidelines are not always suitable for clinical practice in Russia, because of AHD etiology as well as the age of patients undergoing heart valve replacement.

Young women planning pregnancy are considered high risk patients who require careful selection of the optimal prosthetic heart valve. Mechanical heart valve prostheses, which require lifelong indirect anticoagulant therapy (warfarin, phenindione), are not appropriate because of the teratogenic potential of indirect anticoagulants [3, 4]. Current AHA and ESC guidelines should be followed for selecting the type of prosthesis. According to them, the preferable valve choice for this patient group is bioprosthesis [2, 3]. However, bioprosthetic heart valve replacement in young patients requires future reoperations due to the development of bioprosthesis dysfunction. Therefore, alternative options for preservation of xenogenic material are required.

The main issue with bioprosthetic heart valves is their finite lifespan and high risk of reoperation in the future [2]. The average lifespan of mechanical valves is 20 to 30 years, making these valves more suitable for younger patients. In contrast, bioprosthetic heart valves have 8 to 15 years’ durability, depending on patient age, prosthesis type and position [2, 5, 6]. The first successful pregnancy and delivery in a patient with a prosthetic mitral valve (Starr–Edwards) was reported in 1966 [7]. Warfarin embryopathy was first described by Hall in 1965 [7, 8].

A study of pregnant women with prosthetic heart valves in Denmark with a 30-year follow-up confirmed fetal and maternal complications associated with anticoagulant therapy [9]. The true incidence of warfarin-induced embryopathy is difficult to establish. Studies have reported incidences ranging from less than 5 % to more than 67 %; other researchers have estimated that it occurs in 4 to 10 % of cases [10].

Several studies have demonstrated the safety of unfractionated heparin therapy (UFH). Heparin is thought to be an ideal drug in pregnancy, because it does not cross the placenta, and therefore has no effect on the fetus. However, the reliability and efficacy of UFH has not been proven [11–13].

Bioprosthetic heart valves are preferred over mechanical valves in women of childbearing age who are planning pregnancy [2]. Their main advantage is that they allow a better quality of life, mainly because patients do not require anticoagulation therapy (while normal sinus rhythm is maintained in patients in the postoperative period) and the valves have greater resistance to infection. However, bioprosthetic heart valves have less durability than mechanical valves [14]. The risk of structural valve deterioration depends on patient age, with significantly higher risk in young patients. This particular issue determines the selection of the optimal prosthetic heart valve. Thus, approximately 40 % of heart valve implantations in the USA and Europe are bioprosthetic, resulting in higher life expectancy [2]. Bioprosthetic valves are mainly used in older patients with underlying degenerative processes or coronary artery disease. In comparison, only 7 to 10 % of heart valves implanted in younger patients with rheumatic heart diseases in Russia and other developing countries are bioprosthetic, and they carry a relatively high risk of graft dysfunction.

Thus, the main advantage of bioprosthetic heart valves is that most patients avoid anticoagulation therapy. Their significant disadvantage is a high risk of structural valve deterioration in the late postoperative period. Specific complications that limit bioprosthetic durability and result in reoperations in the second decade after implantation include primary tissue degeneration associated with most xenografts, calcification and prosthetic valve endocarditis. If prosthetic valve endocarditis develops as a consequence of non-compliance with preventive therapy, primary tissue degeneration occurs as a separate issue, associated with poorly investigated biological tissue transformations during prosthesis production and the natural-biological function in the recipient. Despite the fact that the problem of bioprosthesis calcification has existed since the earliest models, the pathogenetic mechanisms of xenograft calcification have not been fully investigated. Extensive clinical experience in the application of bioprosthetic heart valves has accumulated worldwide, including different valve models fabricated from different biological tissues and various methods of valve preservation and anti-calcification treatment. Hundreds of thousands of bioprostheses have been implanted and management strategies for patients in the early and late postoperative periods have been developed, but the problem of bioprosthetic heart valve dysfunction remains far from resolved. There is no consensus on bioprostheses in the literature [15]. Sudden mechanical valve failure is usually a fatal event; bioprosthetic failure progresses for years and allows elective reoperation [3].

Several Russian clinics now use new-generation diepoxy-treated bioprostheses. The Russian experience in this area appears to be rare, as no other country has treated xenografts with epoxy compounds; most continue to use glutaraldehyde-treated biomaterials. The hypothesis that the biochemical transformations occurring in xenograft implanted prosthetic devices can be affected by conserving agents has resulted in a new conservation method that increases resistance to biodegradation and calcification. Our experience suggests that ethylene glycol diglycidyl ether, a new-generation conserving agent, improves the structural stability and calcification-resistant properties of bioprosthetic heart valves [15].

We have an 18-year single-center experience with mitral valve replacement using xenoaortic (epoxy-treated) bioprostheses. “KemKor” bioprostheses were implanted in the period from 1991 to 2001 and “PeriCor” prostheses from 2001 to 2009. A total of 382 xenoaortic bioprosthetic heart valves were implanted in the mitral position (the mean age of patients at the time of surgery was 48.8±10.2). The surgeries were performed according to the standard procedure for mitral valve replacement under normothermic cardiopulmonary bypass. The in-hospital mortality was 5.8 % (*n*=22), and 360 patients were successfully discharged from hospital. By the end of 2014, the completeness of patients’ follow-up was 94.5 % with a mean follow-up period of 8.0±4.4 years and a follow-up scope of 2792 patient-years. The long-term mortality, including the redo procedures, was 29.2 % (*n*=105) of the total number of discharged patients. Of the total number of discharged patients, 130 patients (36.1 %) underwent redo procedures because of the development of degenerative changes. Bioprosthetic dysfunction was confirmed by morphological studies in 126 cases (96.9 %). Half of the redo cases (*n*=63) had primary tissue failure with calcification of the xenogenic material, 15.1 % (*n*=19) reported bioprosthetic dysfunction with no signs of calcification, and 27 % (*n*=34) had prosthetic endocarditis. One patient (0.8 %) had prosthetic valve thrombosis, associated with infected tissue prosthesis; nine (7.1 %) patients reported a combination of signs of calcification and infection.

A database was established to allow a detailed analysis of outcomes after KemKor and PeriCor bioprosthetic valve replacements. Below we present the clinical case of a young woman with AHD who received a bioprosthetic heart valve designed in our clinic.

## Case presentation

A 21-year-old Russian woman at 38 weeks’ gestation was admitted to the Regional Maternity Hospital, Kemerovo, Russia in March 1998. She presented in critical condition associated with decompensated heart failure. The first manifestations of the disease had occurred at 28 weeks’ gestation after an acute viral respiratory infection with long-lasting elevated body temperature. Toxic shock syndrome developed, and her heart failure symptoms worsened. Echocardiography (ECHO) revealed infective endocarditis, mitral valve failure (rupture of the anterior mitral valve leaflet with grade IV regurgitation) and mobile vegetation at the posterior leaflet. A healthy baby was delivered by cesarean section at 39 weeks’ gestation. She then received goal-directed therapy in the cardiac surgery unit aimed at suppressing sepsis and compensating for heart failure: initially New York Heart Association (NYHA) class IV. Mitral valve replacement with bioprosthetic heart valve KemKor-30 was performed 6 weeks after delivery. The management of primary infective endocarditis (enterococcal infection verified by blood culture study) and subsequent prevention of prosthetic endocarditis was performed in accordance with the existing (at the time of the follow-up period) guidelines. She received ceftriaxone, 2 g/day administered intravenously in a single daily dose. Her postoperative period was without complications (discharge with NYHA class II). The choice of a bioprosthetic heart valve was determined by her desire for future pregnancy.

Indirect anticoagulant (phenindione) therapy was discontinued 6 months after surgery, because of the absence of heart failure symptoms and cardiac arrhythmias and satisfactory prosthesis function. She became pregnant again in 2002, 3 years after surgery. In the follow-up period, she did not receive any cardiotropic agents and anticoagulants, her condition was satisfactory. Her pregnancy was not contraindicated, according to clinical assessment and testing. She was supervised by surgeons, cardiologists and obstetricians during her pregnancy. She delivered a healthy baby boy vaginally without complications. Her child was breastfed. Annual examinations were performed to monitor for bioprosthetic valve failure, because breastfeeding is known to increase the risk of calcification.

**Fig. 1:**
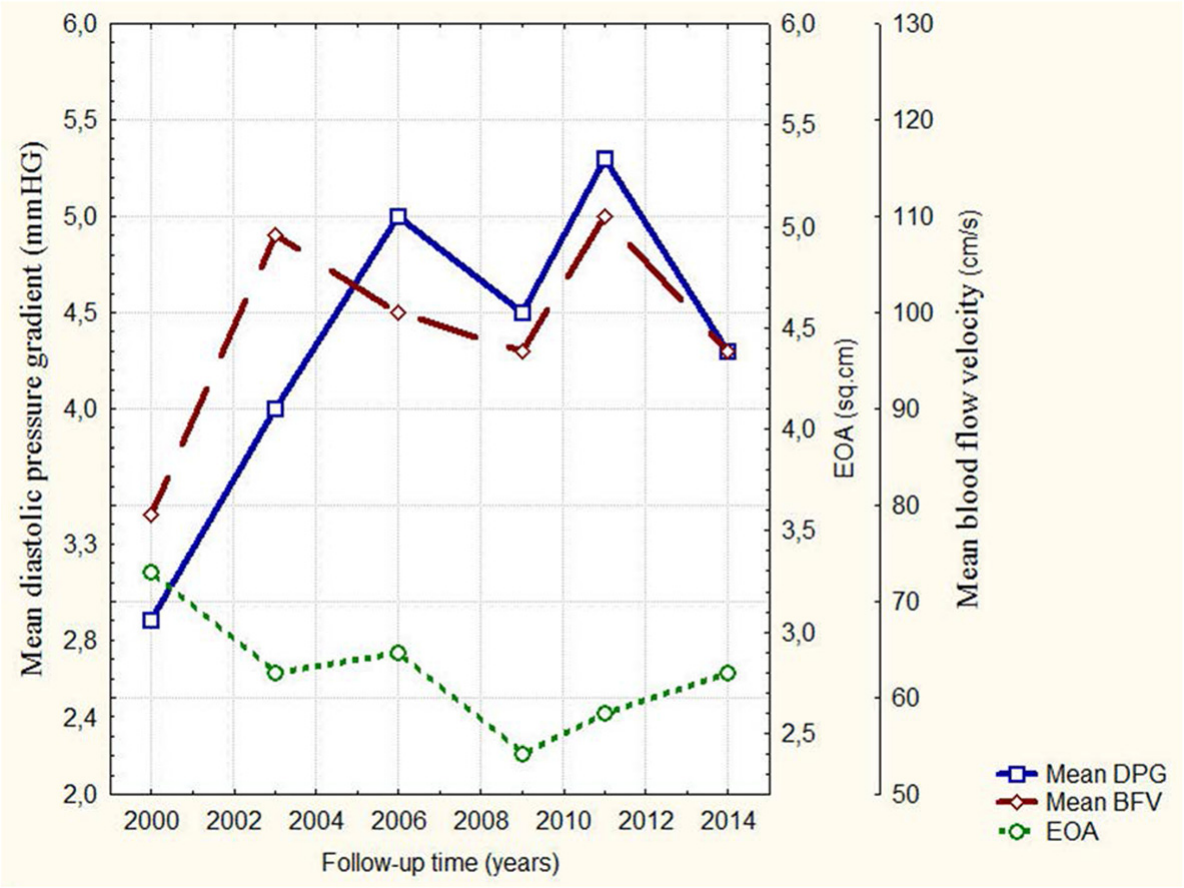
Hemodynamic parameters with the bioprosthesis during follow-up. Mean DPG – mean diastolic pressure gradient, mmHG; Mean BFV – mean blood flow velocity; EOA – effective orifice area

A moderate calcific degeneration of the leaflet was found 12 years after heart valve replacement, a common finding in bioprosthetic heart valves. Dynamic ECHO revealed a moderate decrease in the effective orifice area and an increase in the mean diastolic transprosthetic pressure gradient and mean transprosthetic blood flow velocity. However, these changes still met the criteria for normal prosthetic heart valve function (Fig. 1). She did not receive medical treatment.

She reported pregnancy at 5 weeks’ gestation. Despite the risk of complications associated with limited durability of the prosthetic heart valve and hormonal changes associated with pregnancy and lactation, which could worsen structural valve deterioration, she decided to continue her pregnancy. There were no signs of cardiac arrhythmias on her daily electrocardiograms (ECGs) or signs of heart failure during patient monitoring.

**Fig. 2:**
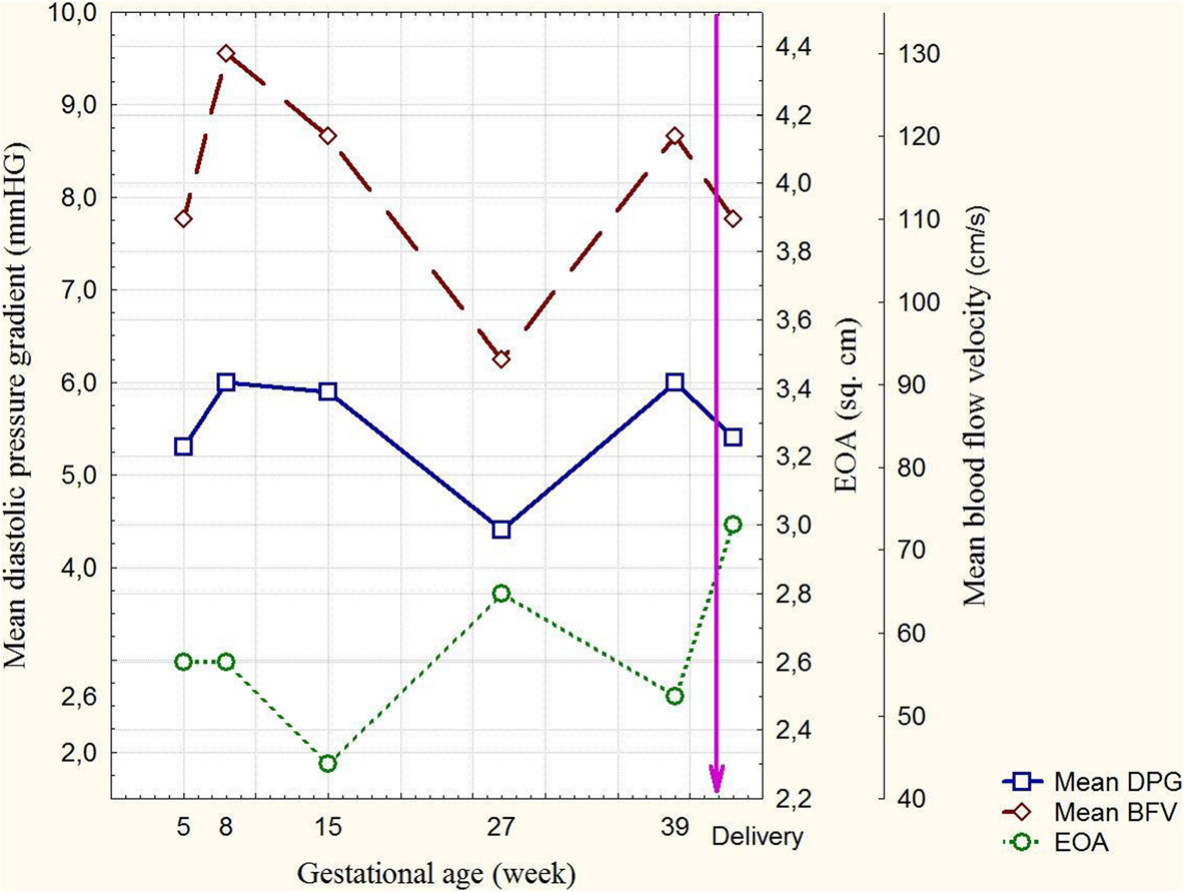
Bioprosthesis hemodynamic parameters during pregnancy. Mean DPG – mean diastolic pressure gradient, mmHG; Mean BFV – mean blood flow velocity; EOA – effective orifice area

Five ECHO examinations revealed the natural physiological changes caused by cardiovascular adaptation to the increased metabolic needs of pregnancy to ensure adequate oxygenated blood delivery to the peripheral tissues and the fetus. The changes included increased circulating blood volume, heart rate, myocardial contractility and systemic blood pressure, which certainly influenced intracardiac hemodynamic parameters [10]. Her left atrium enlarged from 3.4 to 4.0 cm and her left ventricular end-diastolic volume rose from 81 to 99 ml, related to the circulating blood volume and increased preload during pregnancy. A similar mechanism was identified in the central regurgitation across the bioprosthesis registered at 8 weeks’ gestation. The initial transprosthetic regurgitation of > grade I had increased to grade I to II by delivery. However, the absence of significant changes in her diastolic gradient and blood flow velocity confirmed that the prosthesis maintained functional durability (Fig. 2).

Her elevated cardiac output and her left ventricular dilatation were caused by increased myocardial contractility. Her left ventricular ejection fraction rose from 63 to 76 % during her pregnancy. The physiological increase in her heart rate in the last trimester was associated with the overestimation of the effective orifice area. Tachycardia affected the Doppler characteristics of the mitral valve and biased the resulting ECHO parameters. Her mean pulmonary artery pressure increased from 10 mmHg at 5 weeks’ gestation to 16 mmHg at 39 weeks’ gestation, contributing to a moderate increase in peripheral resistance and arterial blood pressure after 32 weeks’ gestation.

The bioprosthesis, implanted in the mitral position, contributed to the physiological changes in functional and morphological parameters of cardiac remodeling, whereas cardiac reserve was not impaired during her pregnancy.

Natural vaginal delivery of a healthy full-term male newborn (3670 kg, 52 cm, Apgar scores 8 to 9) occurred in October 2011, at 39 weeks’ gestation. Her postpartum period was uneventful and she was discharged on the sixth day after delivery.

ECHO findings included reduction of bioprosthesis regurgitation to grade I, reduction of left ventricular end-diastolic volume to 85 mL and increase in mean pulmonary artery pressure to 12 mmHg, indicating stabilization of her intracardiac hemodynamics (Fig. 2).

No significant health problems arose for our patient or her baby during a 2-year follow-up. Her baby was breastfed for 12 months. Our patient did not have any signs of heart failure or clinically significant cardiac arrhythmias. There were no indications for cardiotropic drugs. Dynamic ECHO assessment of the mitral bioprosthesis revealed its functional safety, despite the thickening of its leaflets. Sixteen years after mitral valve replacement, satisfactory left ventricular ejection fraction (64 %), target blood pressure, mean diastolic transprosthetic gradient (4.2 mmHg) and mean transprosthetic flow velocity (96 cm/second) were maintained. The effective orifice area was 2.8 cm^2^. Her left ventricular end-diastolic volume (84 mL) and mean pulmonary artery pressure (13 mmHg) remained stable. Despite grade I regurgitation, the size of her left atrium did not exceed the upper limit of normal (3.9 cm).

## Conclusions

This clinical case demonstrates the use of bioprosthetic heart valves without the need for long-term indirect anticoagulant therapy. Bioprosthetic heart valves are an alternative to mechanical valve prostheses for women of childbearing age. The experience of this young woman with two pregnancies and lactation periods revealed no signs of bioprosthetic valve dysfunction with the diepoxy-treated bioprosthetic valve and supports the use of bioprosthetic heart valves in patients who are planning pregnancies.

Our extensive experience with epoxy-treated bioprosthetic heart valves such as KemKor and PeriCor indicates that patients can avoid long-term anticoagulant therapy because of their optimal hemodynamic functional parameters, thromboresistance and high resistance to infection, without worsening quality of life in the late postoperative period. We recommend these valves both in older persons and in young patients with an active lifestyle, including women who are planning pregnancy.

## Consent

Written informed consent was obtained from the patient for publication of this case report and any accompanying images. A copy of the written consent is available for review by the Editor-in-Chief of this journal.

## Abbreviations

AHA: American Heart Association
AHD: Acquired heart disease
ECHO: Echocardiography
ESC: European Society of Cardiology
NYHA: New York Heart Association
UFH: Unfractionated heparin therapy
